# Pathological response and safety of albumin-bound paclitaxel as a neoadjuvant treatment for HER2-positive breast cancer compared to docetaxel combined with anti-HER2 therapy: a real-world study

**DOI:** 10.3389/fonc.2024.1412051

**Published:** 2024-08-21

**Authors:** Zhidong Lyu, Linlin Gao

**Affiliations:** Breast Surgery, The Affiliated Hospital of Qingdao University, Qingdao, China

**Keywords:** HER2-positive breast cancer, neoadjuvant treatment, albumin-bound paclitaxel, docetaxel, pathological complete response

## Abstract

**Background:**

This study aimed to retrospectively analyse the pathological response and safety of combining albumin-bound paclitaxel (nab-paclitaxel) or docetaxel with anti-HER2 therapy as a neoadjuvant treatment for HER2-positive breast cancer.

**Methods:**

From June 2020 to August 2023, 225 HER2-positive breast cancer patients who underwent radical surgery following neoadjuvant treatment were enrolled in this study. The patients were divided into two groups based on the drugs they received: the nab-paclitaxel group (n=166, receiving nab-paclitaxel + platinum along with trastuzumab and pertuzumab) and the docetaxel group (n=59, receiving docetaxel + platinum along with trastuzumab and pertuzumab). The pathological response and adverse events related to the drugs were collected and evaluated in both groups.

**Results:**

In the nab-paclitaxel group, the rates of breast and total pathological complete response (bpCR and tpCR) were significantly greater than those in the docetaxel group (69.27% vs. 47.45%, P=0.003; 68.67% vs. 45.76%, P=0.002). For patients who did not achieve pCR after chemotherapy, the pathological response of chemotherapy was analysed using MP grading and RCB grading. The results showed that there was a statistically significant difference between the two groups (P<0.05). Multivariate analysis revealed that therapeutic drugs, clinical stage, ER status, and Ki-67 level were independent predictors of pCR. The nab-paclitaxel group had a significantly greater proportion of patients with peripheral sensory neuropathy than did the docetaxel group (58.43% vs. 38.98%, P=0.035), while the docetaxel group had a greater proportion of patients with allergies and elevated ALT (31.93% vs. 69.49%, P=0.000; 23.49% vs. 40.68%, P=0.021).

**Conclusions:**

Our real-world study revealed that nab-paclitaxel combined with anti-HER2 therapy was an effective neoadjuvant therapy for HER2-positive breast cancer. The multivariate analysis revealed that chemotherapy drugs, clinical stage, ER status, and Ki-67 level was the significant factor influencing treatment outcome. These findings offer a valuable reference for the neoadjuvant treatment of patients with HER2-positive breast cancer.

## 1 Introduction

Neoadjuvant therapy is extensively utilized in the treatment of breast cancer, particularly in cases of human epidermal growth factor receptor 2 (HER2)-positive breast cancer and triple-negative breast cancer (1, 2). The advancement of anti-HER2 therapy in recent years has significantly enhanced the overall prognosis for patients with HER2-positive breast cancer (3). In the early NOAH study, a combination of chemotherapy and trastuzumab demonstrated a substantial 21% increase in the pathological complete response (pCR) rate, providing a crucial foundation for neoadjuvant treatment in HER2-positive breast cancer (4). Additionally, another study revealed that the pCR rates were 31%, 23%, and 49% when trastuzumab, pertuzumab, and their combinations were used, respectively (5). The Neosphere trial documented an 18% pCR rate with trastuzumab and pertuzumab, but when combined with docetaxel, the pCR rate increased to 49% (6). These results indicated that the inclusion and/or combination of chemotherapy and anti-HER2 therapy can improve the pCR rate.

Albumin-bound paclitaxel (nab-paclitaxel), a preparation of paclitaxel that binds to albumin, does not require a solvent (7). Studies have shown that when administered at the same dose, nab-paclitaxel reaches tumours at a concentration 1.3 times greater than that of solvent-based paclitaxel, leading to more powerful antitumour effects (8, 9). In a previous report, we observed that nab-paclitaxel was more effective at achieving a pCR rate than docetaxel in patients with HER2-negative breast cancer (10). That study revealed that the pCR rate of patients receiving nab-paclitaxel regimens was 36.71%, which exceeded the rate of 20% achieved with docetaxel regimens. With these positive results, nab-paclitaxel may be considered a new option for combination therapy with anti-HER2 agents.

Trastuzumab is a human monoclonal antibody targeting HER2 that induces antibody-dependent cell-mediated cytotoxicity and inhibits signal transduction (11, 12). Previous studies have shown that neoadjuvant therapy using reference trastuzumab (Herceptin^®^, Roche, USA) has a significant effect on the prognosis of early-stage breast cancer patients (13). Zercepac (HLX02, Fuhong Hanlin Pharmaceutical Co., Ltd., China) is the first Chinese monoclonal antibody highly similar to the reference trastuzumab and has good potential for reducing tumour cell proliferation and survival (14). Recently, Zercepac demonstrated effectiveness equivalent to that of reference trastuzumab for HER2-positive recurrent or metastatic breast cancer in a phase III multicentre clinical trial (15). However, more studies are needed to evaluate its potential effectiveness in neoadjuvant therapy.

In China, nab-paclitaxel has not been used for a long time, and the effectiveness and safety of nab-paclitaxel combined with anti-HER2 agents for treating HER2-positive breast cancer patients need further clinical observation. Consequently, we conducted this real-world study to assess the pathological response and safety of nab-paclitaxel or docetaxel combined with anti-HER2 therapy for the treatment of HER2-positive breast cancer.

## 2 Patients and methods

### 2.1 Patients

**Figure 1:**
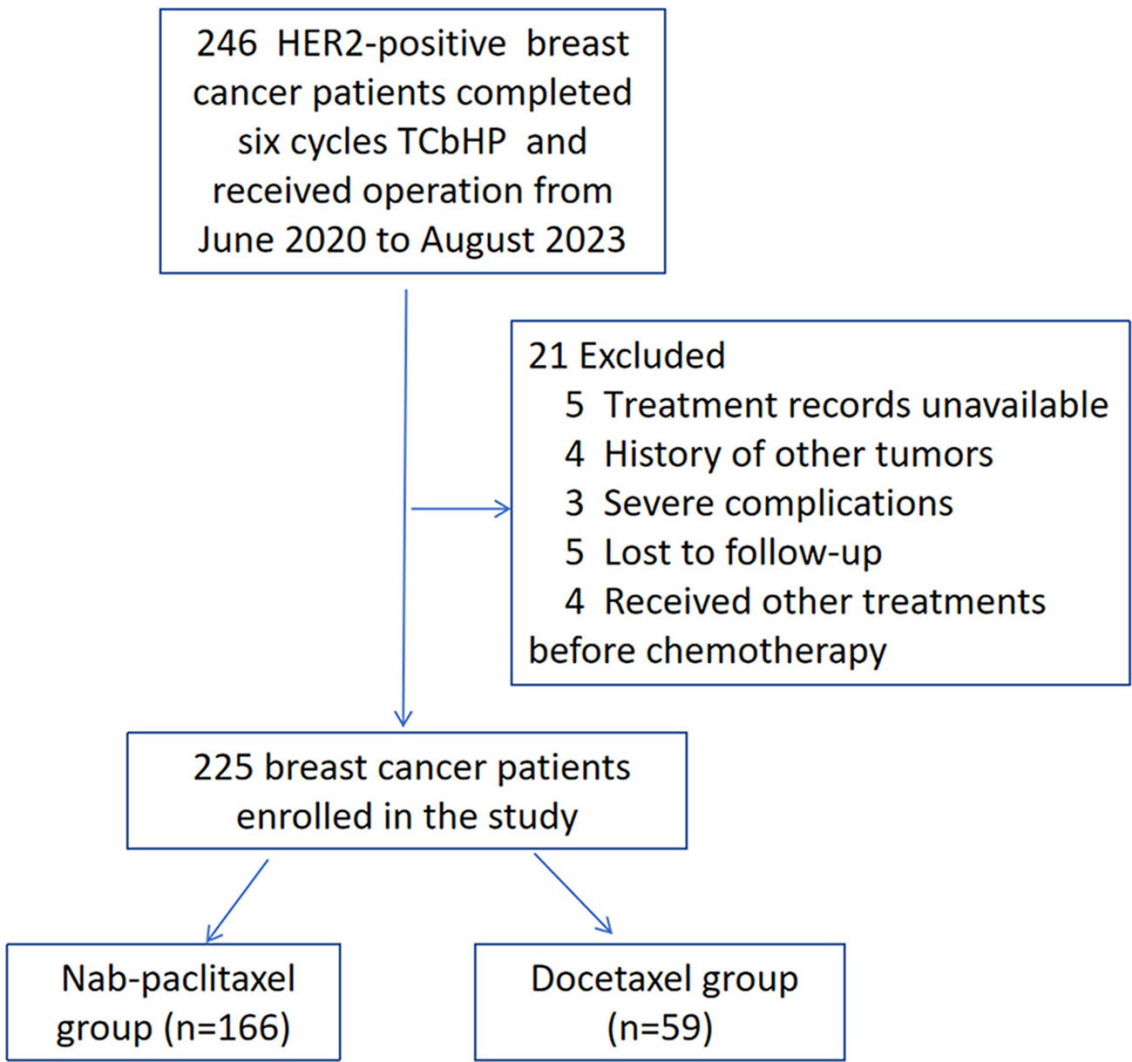
The procedure for screening and grouping patients.

This retrospective study was conducted at the Affiliated Hospital of Qingdao University and focused on HER2-positive breast cancer patients who underwent neoadjuvant treatment followed by radical surgery. The study included patients who met the following criteria: (1) aged ≥18 years, (2) HER2-positive, (3) diagnosed with invasive breast cancer, (4) clinical stage II-III, (5) underwent radical surgery for breast cancer, and (6) received TCbHP therapy (taxane + platinum combined with trastuzumab and pertuzumab, 6 cycles) prior to surgery. Patients who had received any type of therapy before neoadjuvant treatment, had synchronous or previous *in situ* or invasive breast cancer, inflammatory breast cancer, male breast cancer, bilateral breast cancer, chronic or acute inflammatory disease, kidney insufficiency, mental disease, or autoimmune disease were excluded. This study enrolled consecutive patients who met the inclusion criteria from June 2020 to August 2023, resulting in the initial identification of 246 patients. Women who had received other treatments before therapy (n=4), had unavailable treatment records (n=5), had a history of other tumours (n=4), experienced severe complications (n=3), or were lost to follow-up (n=5) were excluded. Finally, the analysis focused on 225 HER2-positive breast cancer patients who were available for analysis (**Figure 1**). Patients were categorized into either the docetaxel group (n=59) or the nab-paclitaxel group (n=166) based on the medication they received. The treatment involved administering either nab-paclitaxel (Qilu Pharmaceutical Co., Ltd., China) at a dose of 260 mg/m^2^ or docetaxel (Kelun Pharmaceutical Co., Ltd., China) at a dose of 75 mg/m^2^, along with carboplatin (Qilu Pharmaceutical Co., Ltd., China) at the AUC5. Additionally, all patients received trastuzumab at a dose of 8 mg/kg in the first cycle, 6 mg/kg in subsequent cycles, and pertuzumab (Perjeta, Roche, USA) at a dose of 840 mg in the first cycle and 420 mg g in subsequent cycles every 3 weeks. The physicians determined the specific doses and schedules for the treatment based on the diagnosis and treatment guidelines. Subsequently, the patients underwent radical surgery 3-4 weeks after completing the last neoadjuvant treatment.

Before starting neoadjuvant treatment, a core needle biopsy was performed to obtain a pathological diagnosis. Immunohistochemistry was used to evaluate the levels of estrogen receptor (ER), progesterone receptor (PR), HER2, and Ki-67. A positive expression of ER or PR was defined as a rate of 1% or higher. According to the ASCO/CAP guidelines, HER2 positivity was defined as a score of 3+ on immunohistochemistry or a score of 2+ on FISH amplification. The Ki-67 level was calculated as the percentage of tumour cells showing positive nuclear staining. The classification of ki-67 in this article relies on St. Gallen consensus (16). This study was approved by the Medical Ethics Review Board of the Affiliated Hospital of Qingdao University (QYFY WZLL 28131). It should be noted that this study was a retrospective analysis, and the ethics committee waived the need for informed consent. Patient information has been anonymized in this article to protect privacy.

### 2.2 Pathological response and toxicity assessments

Evaluations of the effectiveness of neoadjuvant treatment were conducted every 2 cycles using ultrasonography or MR imaging. The pathological reports were independently reviewed by 2 pathologists to determine the category. The response to neoadjuvant treatment was assessed according to RECIST version 1.1. Total pathological complete response (tpCR) was defined as the absence of any pathological evidence of residual invasive carcinoma in both the breast and axillary lymph nodes (ypT0/isN0 status). A breast pathological complete response (bpCR) was defined as the absence of any pathological evidence of residual invasive carcinoma in the breast (ypT0/is status). The Miller-Payne (MP) system, which consists of 5 severity grades, was employed in the study to assess the degree of tumour reduction, with higher grades indicating a greater reduction. Grade 5 corresponds to a complete pathological response in breast cancer (17). The pathological response of treatment was also analysed using the residual cancer burden (RCB) grading system, which involves classification into levels 0-3 based on guidelines from the International Breast Collaboration Group (18). Adverse events were collected and graded using CTCAE 5.0.

### 2.3 Statistical analysis

The primary endpoint of this study was the PCR. The comparisons of clinical and pathological characteristics were made using Fisher’s exact test or Pearson’s chi-square test. Logistic regression models were used for both univariate and multivariate analyses. We first conducted a single factor analysis, and then conducted the multivariate logistic analysis based on the differences identified from the single factor analysis(P<0.05). Data analysis was performed using SPSS software version 19.0, with a significance level of P<0.05.

## 3 Results

### 3.1 Basic patient characteristics

This study analysed a total of 225 patients, with 166 receiving nab-paclitaxel-based treatment and 59 receiving docetaxel-based regimens. The baseline characteristics of all patients are presented in **Table 1**. The average age of the patients in the nab-paclitaxel group was 49.49 ± 8.88 years, while that in the docetaxel group was 51.76 ± 12.83 years. The two groups had similar basic features, including age (P=0.213), sides (P=0.244), menstruation status (P=0.363), tumour size (P=0.555), histological grade (P=0.261), lymph node status (P=0.914), clinical stage (P=0.057), ER status (P=0.689), PR status (P=0.986), PR status (P=0.635), Ki-67 level (P=0.292), and targeted therapy (P=0.165).

**Table 1:** Basic characteristics of patients.

Patients characteristics	Total (n=225)	Nab-paclitaxel (n=166)	Docetaxel (n=59)	χ2	*P*
Age(years), mean ± SD	50.09 ± 10.09	49.49 ± 8.88	51.76 ± 12.83	**t=-1.255**	0.213
Age(years)					
≤50	111	86	25	1.550	0.213
>50	114	80	34		
Sides					
Left	110	85	25	1.359	0.244
Right	115	81	34		
Menstruation situation					
Premenopausal	116	89	27	1.075	0.363
Postmenopausal	109	77	32		
Tumor size					
≤3cm	62	44	18	0.349	0.555
>3cm	163	122	41		
Histological grade					
I	9	8	1	0.269	0.261
II	179	134	45		
III	37	24	13		
Lymph node status					
Negative	14	11	3	0.012	0.914
Positive	211	155	56		
Clinical stage					
II	76	62	14	3.61	0.057
III	149	104	45		
ER status					
Negative	117	85	32	0.160	0.689
Positive	108	81	27		
PR status					
Negative	107	79	28	0.000	0.986
Positive	118	87	31		
HER2 status					
IHC 2+, FISH+	16	10	6	0.225	0.635
IHC 3+	209	156	53		
Ki-67 level					
≤20%	50	34	16	1.109	0.292
>20%	175	132	43		
Targeted medicine					
Zercepac	86	59	27	1.926	0.165
Reference Trastuzumab	139	107	32		

### 3.2 Pathological response

Following neoadjuvant therapy, the bpCR rate in the nab-paclitaxel group was 69.27% (115/166), which was significantly greater than the 47.45% (28/59) in the docetaxel group (P=0.003). Similar findings were observed for tpCR, for which the tpCR rate was 68.67% (114/166) in the nab-paclitaxel group, which was significantly greater than the 45.76% (27/59) in the docetaxel group (P=0.002). Subgroup analysis revealed that among patients with a tumour size >3 cm, the nab-paclitaxel group achieved higher rates of bpCR and tpCR than did the docetaxel group (P<0.05). Additionally, subgroup analysis revealed that in the nab-paclitaxel group, patients with a positive lymph node grade, clinical stage III disease, ER-negative status, PR negative status, HER2 status, IHC 3+ status, and Ki67 ≤ 20% achieved a significantly greater pCR rate than did those receiving docetaxel (P<0.05) (**Table 2**). Furthermore, when comparing the pathological response of reference trastuzumab and Zercepac combined with taxane in treating HER2-positive breast cancer, both reference trastuzumab and Zercepac combined with nab-paclitaxel resulted in higher tpCR rates than did treatment combined with docetaxel alone (69.15% vs. 46.88%, P=0.040; 69.80% vs. 44.44%, P=0.021).

**Table 2:** Comparison of pathological complete response rates between the both groups.

	bpCR					tpCR				
Patients characteristics	Nab-paclitaxel (n=166)	Docetaxel (n=59)	OR	95%CI	*P*	Nab-paclitaxel (n=166)	Docetaxel (n=59)	OR	95%CI	*P*
All	115/166	28/59	0.401	0.218-0.736	0.003	114/166	27/59	0.385	0.209-0.707	0.002
Age(years)										
≤50	65/86	12/25	0.538	0.276-1.051	0.068	64/86	12/25	0.573	0.295-1.112	0.098
>50	50/80	16/34	0.963	0.342-2.708	0.943	50/80	15/34	0.855	0.303-2.411	0.767
Tumor size										
≤3cm	34/44	10/18	0.368	0.114-1.181	0.087	33/44	9/18	1.333	0.477-3.728	0.583
>3cm	81/122	18/41	0.396	0.192-0.816	0.011	81/122	18/41	0.396	0.192-0.816	0.011
Histological grade										
I	7/8	1/1	1.143	0.880-1.485	0.724	7/8	1/1	1.143	0.880-1.485	0.724
II	92/134	22/45	0.437	0.219-0.870	0.017	91/134	21/45	0.413	0.208-0.823	0.011
III	16/24	5/13	0.313	0.077-1.271	0.103	16/24	5/13	0.313	0.077-1.271	0.103
Lymph node grade										
Negative	7/11	2/3	1.143	0.077-16.947	0.925	7/11	2/3	1.143	0.077-16.947	0.925
Positive	108/155	26/56	0.377	0.201-0.706	0.002	107/155	25/56	0.362	0.193-0.677	0.001
Clinical stage										
II	47/62	8/14	0.426	0.127-1.424	0.280	47/62	7/14	0.319	0.096-1.058	0.172
III	68/104	20/45	0.424	0.208-0.864	0.017	67/104	20/45	0.442	0.217-0.900	0.023
ER status										
Negative	70/85	17/32	0.243	0.100-0.592	0.001	69/85	17/32	0.263	0.109-0.635	0.002
Positive	45/81	11/27	0.550	0.227-1.331	0.182	45/81	10/27	0.471	0.192-1.152	0.096
PR status										
Negative	66/79	15/28	0.227	0.088-0.589	0.001	65/79	15/28	0.249	0.097-0.637	0.003
Positive	49/87	13/31	0.560	0.244-1.284	0.168	49/87	12/31	0.490	0.212-1.132	0.092
HER2 status										
IHC 2+, FISH+	4/10	1/6	0.300	0.025-3.626	0.345	4/10	1/6	0.300	0.025-3.626	0.345
IHC 3+	111/156	27/53	0.421	0.222-0.799	0.007	110/156	26/53	0.403	0.213-0.763	0.005
Ki-67 level										
≤20%	21/34	3/16	0.143	0.034-0.599	0.005	20/34	2/16	0.100	0.020-0.511	0.002
>20%	94/132	25/43	0.561	0.275-1.146	0.110	94/132	25/43	0.561	0.275-1.146	0.110
Targeted medicine										
Zercepac	41/59	13/27	0.408	0.160-1.040	0.057	40/59	12/27	0.380	0.149-0.968	0.040
Reference Trastuzumab	74/107	15/32	0.393	0.176-0.881	0.021	74/107	15/32	0.393	0.176-0.881	0.021

**Figure 2:**
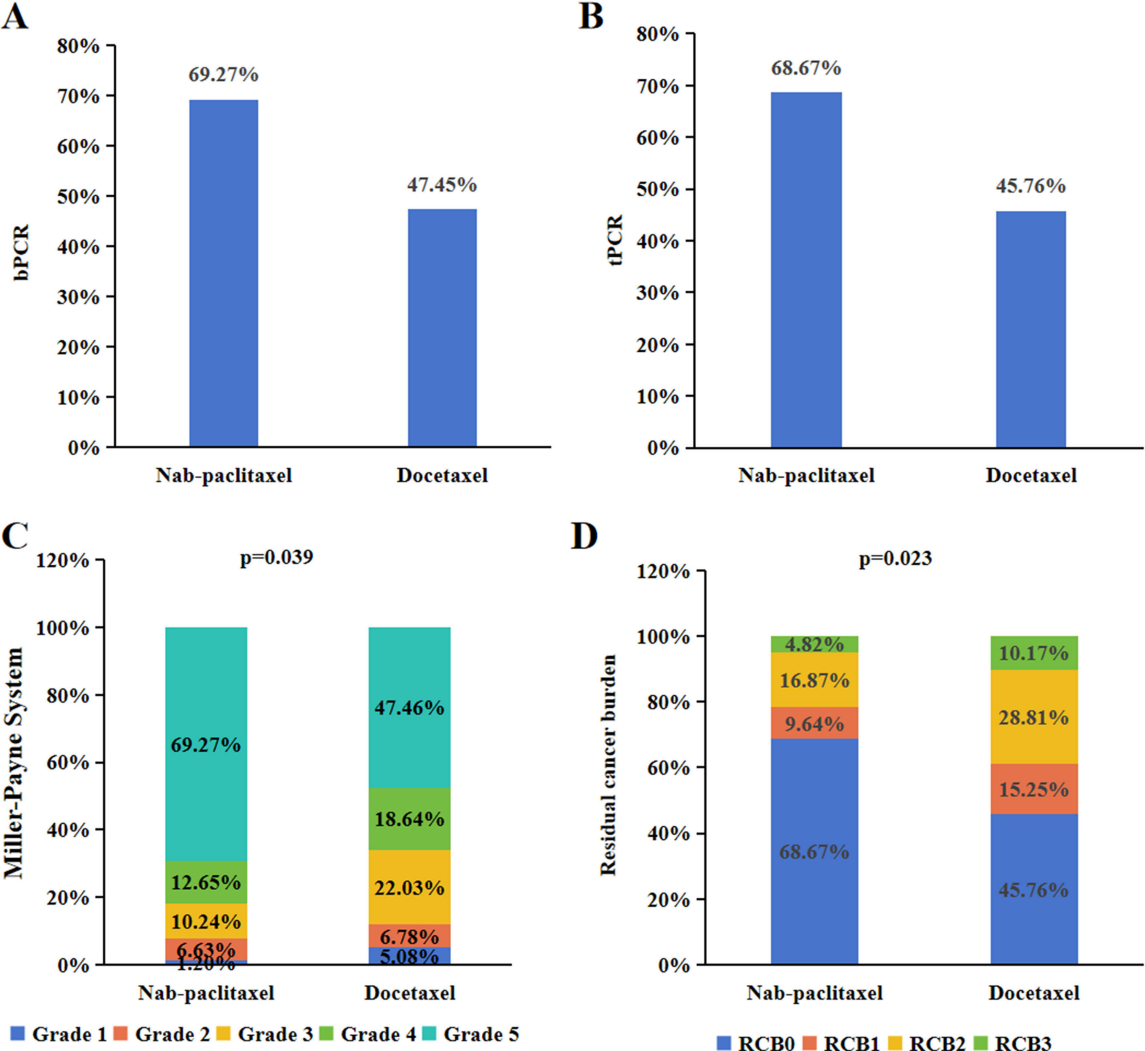
Pathological response of neoadjuvant treatment for HER2-positive breast cancer. The bpCR **(A)** and tpCR **(B)** of nab-paclitaxel or docetaxel combination with anti-HER2-therapy in HER2-positive breast cancer. The MP grading **(C)** and RCB grading **(D)** of nab-paclitaxel or docetaxel combination with anti-HER2-therapy in HER2-positive breast cancer.

To assess the pathological response of chemotherapy in patients who did not achieve pCR, we utilized MP grading and RCB grading. In the nab-paclitaxel group, Grade 1 partial response occurred in 1.20% of patients, Grade 2 partial response in 6.63%, Grade 3 partial response in 10.24%, and Grade 4 partial response in 12.65%. For the docetaxel group, Grade 1 partial response was reported in 5.08% of patients, Grade 2 in 6.78%, Grade 3 in 22.03%, and Grade 4 in 18.64% among HER2-positive patients, and this difference was found to be statistically significant (P=0.039). Additionally, RCB analysis was conducted to evaluate the breast masses and axillary lymph nodes postchemotherapy. In the nab-paclitaxel group, RCB 1 partial response was observed in 9.64% of patients, RCB 2 in 16.87%, and RCB 3 in 4.82%. For the docetaxel group, RCB 1 partial response was reported in 15.25% of HER2-positive patients, RCB 2 in 28.81%, and RCB 3 in 10.17% (**Figure 2**), showing a statistically significant difference (P=0.023).

### 3.3 Outcome associated with pCR

The bpCR rate was 63.56% (143/225), and the tpCR rate was 62.67% (141/225). The univariate logistic analysis results showed that chemotherapy drugs, clinical stage, ER status, PR status and Ki-67 level were associated with bpCR and tpCR (all P<0.05, **Tables 3**, **4**). We then conducted the multivariate logistic analysis based on the differences identified from the single factor analysis. The outcomes of the multivariate logistic regression analysis for bpCR and tpCR are shown in **Tables 5**, **6**. Multivariate analysis revealed that the nab-paclitaxel group exhibited significantly improved bpCR and tpCR compared with the docetaxel group (P<0.05). The results indicated that chemotherapy drugs, clinical stage, ER status, PR status, and Ki-67 level were independent predictors of bpCR (all P<0.05). Furthermore, the multivariate analysis revealed that chemotherapy drugs, clinical stage, ER status, and Ki-67 level was the significant factor influencing tpCR (all P<0.05).

**Table 3:** Univariate analysis of bpCR according to subgroups.

Variable	Non-bpCR (n=82)	bpCR (n=143)	OR	95% CI	P
Age(years)					
≤50	34	77	0.607	0.351-1.051	0.075
>50	48	66			
Tumor size					
≤3cm	18	44	1.098	0.481-2.506	0.824
>3cm	64	99			
Histological grade					
I-II	66	122	0.710	0.347-1.453	0.349
III	16	21			
Lymph node status					
Negative	5	9	0.682	0.207-2.248	0.529
Positive	77	134			
Clinical stage					
II	21	55	0.501	0.273-0.918	0.025
III	61	88			
ER status					
Negative	30	87	0.342	0.195-0.601	0.000
Positive	52	56			
PR status					
Negative	26	81	0.326	0.184-0.580	0.000
Positive	56	62			
HER2 status					
IHC 2+, FISH+	11	5	1.824	0.658-5.060	0.248
IHC 3+	71	138			
Ki-67 level					
≤20%	26	24	0.483	0.255-0.914	0.025
>20%	56	119			
Chemotherapy drugs					
Nab-paclitaxel	51	115	0.401	0.218-0.736	0.003
Docetaxel	21	28			
Targeted medicine					
Zercepac	32	54	1.144	0.655-1.937	0.637
Reference Trastuzumab	50	89			

**Table 4:** Univariate analysis of tpCR according to subgroups.

Variable	Non-tpCR (n=84)	btCR (n=141)	OR	95% CI	P
Age(years)					
≤50	35	76	0.611	0.354-1.054	0.077
>50	49	65			
Tumor size					
≤3cm	20	42	0.737	0.397-1.367	0.333
>3cm	64	99			
Histological grade					
I-II	68	120	0.755	0.364-1.521	0.417
III	16	21			
Lymph node status					
Negative	5	9	0.655	0.199-2.158	0.487
Positive	79	132			
Clinical stage					
II	22	54	0.521	0.286-0.948	0.033
III	62	87			
ER status					
Negative	31	86	0.345	0.197-0.604	0.000
Positive	53	55			
PR status					
Negative	27	80	0.332	0.188-0.587	0.000
Positive	57	61			
HER2 status					
IHC 2+, FISH+	11	5	1.750	0.631-4.852	0.282
IHC 3+	73	136			
Ki-67 level					
≤20%	28	22	0.411	0.217-0.780	0.007
>20%	56	119			
Chemotherapy drugs					
Nab-paclitaxel	52	114	0.385	0.209-0.707	0.002
Docetaxel	32	27			
Targeted medicine					
Zercepac	34	52	1.261	0.725-2.192	0.412
Reference Trastuzumab	50	89			

**Table 5:** Multivariate analysis of bpCR.

Variable	Effect	OR	95% CI	P
Chemotherapy drugs	Nab-paclitaxel vs. Docetaxel	0.403	0.206-0.786	0.008
Clinical stage	II vs. III	0.415	0.211-0.816	0.011
ER status	Negative vs. Positive	0.383	0.192-0.766	0.007
PR status	Negative vs. Positive	0.493	0.246-0.987	0.046
Ki-67 level	>20% vs. ≤20%	0.431	0.208-0.894	0.024

**Table 6:** Multivariate analysis of tpCR.

Variable	Effect	OR	95% CI	P
Chemotherapy drugs	Nab-paclitaxel vs. Docetaxel	0.385	0.197-0.754	0.005
Clinical stage	II vs. III	0.424	0.216-0.832	0.013
ER status	Negative vs. Positive	0.371	0.185-0.743	0.005
PR status	Negative vs. Positive	0.513	0.256-1.013	0.059
Ki-67 level	>20% vs. ≤20%	0.351	0.168-0.733	0.005

### 3.4 Adverse events

Safety as the second endpoint of this study. All patients enrolled in the study completed 6 cycles of TCbHP therapy within both groups. The drug-related adverse events were mild and are listed in **Table 7**. The most commonly observed adverse reaction during neoadjuvant treatment was fatigue, with an incidence of 68.67% in the nab-paclitaxel group and 59.32% in the docetaxel group. However, there was no statistically significant difference between the two groups (P=0.154). Peripheral sensory neuropathy was more prevalent in the nab-paclitaxel group (58.43% vs. 38.98%, P=0.035), whereas the incidence of allergies in this group was lower than that in the docetaxel group (31.93% vs. 69.49%, P=0.000). Additionally, the occurrence of elevated ALT was significantly lower in the nab-paclitaxel group than in the docetaxel group (23.49% vs. 40.68%, P=0.021). Other drug-related adverse events, such as diarrhoea, neutropenia, vomiting, dizziness, intermittent fever, infusion-related reactions, decreased appetite, elevated AST, anaemia, oral mucositis, and difficulty breathing were similar in both groups (P>0.05).

**Table 7:** Treatment-related adverse events.

Toxicity	Nab-paclitaxel (n=166)	Docetaxel (n=59)	χ^2^	*P*
Diarrhea				
0	79	29	0.825	0.662
1~2	73	23		
3~4	14	7		
Neutropenia				
0	92	34	0.454	0.797
1~2	55	17		
3~4	19	8		
Vomit				
0	86	26	1.065	0.587
1~2	67	28		
3~4	13	5		
Fatigue				
0	52	24	3.739	0.154
1~2	80	29		
3~4	34	6		
Dizziness				
0	118	50	5.323	0.070
1~2	41	9		
3~4	7	0		
Hectic fever				
0	113	44	3.154	0.207
1~2	45	15		
3~4	8	0		
Infusion-related reaction				
0	154	54	2.442	0.295
1~2	9	5		
3~4	3	0		
Decreased appetite				
0	101	41	1.511	0.470
1~2	53	14		
3~4	12	4		
Elevated AST				
0	123	43	5.927	0.052
1~2	23	14		
3~4	20	2		
Elevated ALT				
0	127	35	7.762	0.021
1~2	26	19		
3~4	13	5		
Anemia				
0	136	43	2.730	0.255
1~2	25	12		
3~4	5	4		
Allergy				
0	113	18	44.142	0.000
1~2	48	33		
3~4	5	8		
Peripheral sensory neuropathy				
0	69	36	6.713	0.035
1~2	75	17		
3~4	22	6		
Oral mucositis				
0	92	30	4.190	0.123
1~2	50	25		
3~4	24	4		
Insomnia				
0	120	54	9.648	0.008
1~2	29	2		
3~4	17	3		
Difculty breathing				
0	144	51	4.729	0.094
1~2	13	8		
3~4	9	0		

## 4 Discussion

The standard neoadjuvant treatment option for HER2-positive breast cancer has become the combination of chemotherapy and anti-HER2 therapy (19, 20). Nab-paclitaxel shows superior effects in advanced breast cancer and advantages in neoadjuvant therapy (8). The objective of this retrospective study was to evaluate the pathological response and safety of nab-paclitaxel or docetaxel combined with anti-HER2 therapy for the treatment of HER2-positive breast cancer.

The GeparSepto trial demonstrated that weekly nab-paclitaxel is more effective than solvent-based paclitaxel followed by cyclophosphamide plus epirubicin as neoadjuvant therapy in breast cancer, as it significantly improves the pCR rate and disease-free survival (21, 22). It is believed that the albumin-mediated delivery of nab-paclitaxel may enhance its transportation to tumours, improve tolerability, reduce infusion time, and eliminate the need for preoperative prophylactic medication. In a trial involving metastatic breast cancer patients, patients treated with nab-paclitaxel had a greater response rate and longer progression time than patients treated with paclitaxel (23, 24). Previous studies have shown that the safety of nab-paclitaxel is acceptable, but there is a lack of direct comparisons between nab-paclitaxel and docetaxel. The aim of this study was to evaluate the clinical benefits and adverse events of nab-paclitaxel as a neoadjuvant therapy in HER2-positive breast cancer patients. In the present study, we found that nab-paclitaxel combined with anti-HER2 therapy resulted in higher pCR rates than docetaxel group. For all patients, the bpCR rate was 63.56%, and the tpCR rate was 62.67%. We also noticed that the PCR rate in the current study was lower than that in previous studies (TRAIN-2 and TRYPHAENA). In the TRAIN-2 study, a pCR rate of 68% was achieved after 9 cycles of TCbHP treatment (24). The TRYPHAENA study also confirmed a pCR rate of 66.2% after 6 cycles of TCbHP treatment (25). In the present study, the patients enrolled generally had late clinical stage disease, and 93.8% of patients had lymph node metastasis. The proportion of PCR products is also related to the number of cycles of chemotherapy. In this study, patients received 6 cycles of chemotherapy, while patients received 9 cycles of chemotherapy in the TRAIN-2 study (23). Neosphere and Peony utilized the THP regimen for HER2-positive breast cancer and reported a lower pCR rate of only 39.3% (6, 26). These studies further support the important role of platinum drugs in the treatment of HER2-positive breast cancer. Our findings additionally indicated that therapeutic drugs, clinical stage, ER status, and Ki-67 level were all independent predictors of pCR. Ki-67 is an indicator of tumour proliferation, and we found that Ki-67>20% resulted in a higher bPCR rate than Ki-67 ≤ 20% (68.0% vs. 48.0%) in this study. We further found that patients with Ki-67>20% benefitted more from nab-paclitaxel than those with Ki-67 ≤ 20% (71.2% vs. 61.76%). Furthermore, we compared the effectiveness of domestically produced Zercepac with that of the reference trastuzumab. Our data demonstrated that the pCR of HER2-positive breast cancer patients treated with nab-paclitaxel combined with reference trastuzumab was comparable to that of patients treated with Zercepac.

Previous studies have shown that nab-paclitaxel is associated with a greater incidence of peripheral sensory neuropathy, clinical safety and adverse reactions (27, 28). In contrast to docetaxel, nab-paclitaxel does not utilize nonionic surfactants to dissolve paclitaxel. These surfactants are known to cause toxicity and encapsulate paclitaxel in solvent-based micelles. This could be due to the unaffected delivery of nab-paclitaxel by solvents, as higher doses can be administered in comparison to docetaxel (29). TCbHP is a chemical treatment that is accompanied by severe side effects, with leukopenia being the most commonly observed adverse reaction. Furthermore, allergies and nausea are also frequent adverse reactions. The toxicity characteristics observed in this study closely resemble those found in the GeparSepto and ETNA studies (23). The nab-paclitaxel group had a greater incidence of peripheral sensory neuropathy, while the docetaxel group was more prone to allergies. In the GeparSepto study, adjusting the dose of nab-paclitaxel from 150 mg/m^2^ to 125 mg/m^2^ resulted in a decrease in the incidence of grade 3-4 peripheral sensory neuropathy from 15% to 8% in the nab-paclitaxel group (30). Moreover, other drug-related adverse events, such as nausea, joint pain, and difficulty breathing, were similar between the both groups.

This study has several potential limitations that should be considered. First, this was a retrospective study within a single institution, and the sample size was relatively small. We have predicted the power of the analysis using STATA software. When the α value is set to 0.1, the power of the analysis can reach 0.8. We believe this can meet the sample size required for the study. Second, due to its expensive price, nab-paclitaxel did not receive widespread application in the early stage. After the launch of domestically produced drugs in China, nab-paclitaxel have been widely used, our cases were mainly included in 2022 and 2023, this study did not include follow-up data or analyse long-term survival rates. In the future, larger samples and longer follow-up periods are needed to verify these results.

In conclusion, our real-world study revealed that nab-paclitaxel combined with anti-HER2 therapy was an effective neoadjuvant therapy for HER2-positive breast cancer. The multivariate analysis revealed that clinical stage, ER status, and Ki-67 status was the significant factor influencing treatment outcome. The results provide a valuable reference for the neoadjuvant treatment of patients with HER2-positive breast cancer.

## Data Availability

The raw data supporting the conclusions of this article will be made available by the authors, without undue reservation.
